# Comparison of Merging and Meta-Analysis as Alternative Approaches for Integrative Gene Expression Analysis

**DOI:** 10.1155/2014/345106

**Published:** 2014-01-12

**Authors:** Jonatan Taminau, Cosmin Lazar, Stijn Meganck, Ann Nowé

**Affiliations:** Computational Modeling Lab, Vrije Universiteit Brussel, 1050 Brussels, Belgium

## Abstract

An increasing amount of microarray gene expression data sets is available through public repositories. Their huge potential in making new findings is yet to be unlocked by making them available for large-scale analysis. In order to do so it is essential that independent studies designed for similar biological problems can be integrated, so that new insights can be obtained. These insights would remain undiscovered when analyzing the individual data sets because it is well known that the small number of biological samples used per experiment is a bottleneck in genomic analysis. By increasing the number of samples the statistical power is increased and more general and reliable conclusions can be drawn. In this work, two different approaches for conducting large-scale analysis of microarray gene expression data—meta-analysis and data merging—are compared in the context of the identification of cancer-related biomarkers, by analyzing six independent lung cancer studies. Within this study, we investigate the hypothesis that analyzing large cohorts of samples resulting in merging independent data sets designed to study the same biological problem results in lower false discovery rates than analyzing the same data sets within a more conservative meta-analysis approach.

## 1. Introduction

Nowadays, an increasing amount of gene expression data sets is available through public repositories (e.g., NCBI GEO [1], ArrayExpress [2]), which might contain the necessary clues for the discovery of new findings, leading to the development of new treatments or therapies. It is one of the most recent challenges to unlock the hidden potential of these data, by using it in large-scale analysis pipe-lines. Integrating this vast amount of data originating from different but independent studies could be beneficial for the discovery of new biological insights by increasing the statistical power of gene expression analysis [3, 4].

**Figure 1:**
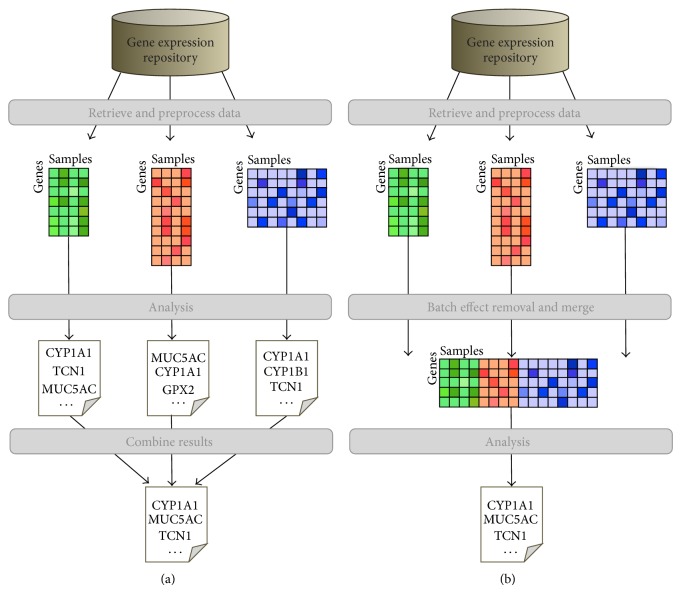
Schematic overview of the two main approaches of integrative microarray analysis in the context of the identification of differential genes (DEGs). (a) Meta-analysis first derives results from each individual study and then combines the results. (b) Merging first combines the data and then derives a result from this large data set.

With *integrative analysis* we mean combining the information of multiple and independent studies, designed to study the same biological problem, in order to extract more general and more reliable conclusions. To this purpose, two approaches exist: *meta-analysis* and *analysis by data merging*. In the meta-analysis approach the results of individual studies (e.g., *P* values, ranks, classification accuracies, etc.) are combined at the interpretative level. In contrast, the merging approach integrates microarray data at the expression value level after transforming the expression values to numerically comparable measures. Both approaches are illustrated in Figure 1. The first step, selecting and retrieving all appropriate data sets, is the same for both scenarios. The main difference between meta-analysis and the integrative analysis via data merging is visible in the next two steps: according to the meta-analysis approach (Figure 1(a)) each data set is analyzed individually and the final results are combined, while according to the merging approach (Figure 1(b)) the data sets are first combined in a unique, much larger data set and then analyzed.

The benefits of integrating multiple microarray gene expression studies are straightforward. Combining information from multiple existing studies can increase the reliability and generalizability of results. Through the integrative analysis of microarray data the sample size increases and with it the statistical power to obtain a more precise estimate of gene expression results. This immediately overcomes the problem of low sample sizes, which is the main limitation for individual microarray studies. At the same time the heterogeneity of the overall estimate is assessed, making the results more generalizable. This way we avoid the danger of study-specific findings or artifacts. Integrative analysis is also a relatively easy and inexpensive way of gaining new biological insights since it makes comprehensive use of already available data accumulated through the years by various groups all over the world.

In [5, 6] several issues for integrative analysis are reported, and while being specific for meta-analysis, many of them are also relevant for merging. Most of these issues are however related to the retrieval and preprocessing of the data and can be solved by using an appropriate data acquisition approach, as we show later in this work (see Section 2). For the merging approach an additional issue has to be mentioned. Before combining the expression values of different studies, they have to be made *comparable* to each other. Since the use of different experimentation plans, platforms, and methodologies by different research groups introduces undesired batch effects in the gene expression values [7, 8] an additional transformation of the data to remove those effects is needed.

To the best of our knowledge no real comparison of both approaches can be found in the literature. In [9] a merging approach is preferred to find a robust prognostic marker for breast cancer using multiple microarray data sets, based on the hypothesis that “*[…] deriving separate statistics and then averaging is often less powerful than directly computing statistics from aggregated data.*” There is however no empirical evidence to validate this statement yet.

In this work we investigate both approaches in the context of the identification of lung cancer-related differentially expressed genes (DEGs) or biomarkers, that is, genes that have a discriminating profile in normal tissues versus lung cancer tissues. Those genes can provide new insights into the biological mechanisms of lung cancer and of cancer in general and can lead to promising biomarkers and new directions for drug development or treatments. We select a number of already published and publicly available data sets containing both normal and cancer tissues and identify robust and stable DEGs using both approaches for integrative analysis.

## 2. Material and Methods

### 2.1. Data Acquisition

To retrieve relevant publicly available data sets we used the InSilico DB [10] as a starting point. InSilico DB is a web-based central warehouse containing ready-to-use, consistently preprocessed, and expert-curated genome-wide data sets (https://insilicodb.org/app/). A list of potential data sets was programmatically retrieved from the InSilico DB using the getDatasetList function from the R/Bioconductor inSilicoDb package [11]. This list was further restricted by defining the following constraints.

- Only frozen RMA (fRMA, [12]) processed studies were considered, that is, studies for which the original CEL files were available and which were consistently preprocessed by the internal InSilico genomic pipeline.
- Each study should contain at least 30 samples in order to be statistically relevant.
- Each study should contain both samples from normal tissue and from lung cancer tissue, more or less equally distributed. In order to achieve this we looked at the “Disease” keyword which is available in most curations and filtered on “lung cancer” | “adenocarcinoma” values and “control” | “normal” | “healthy” values for lung cancer and normal samples, respectively.
- Only studies assayed on Affymetrix Human Genome U133A (GPL96) and Affymetrix Human Genome U133 Plus 2.0 (GPL570) were taken into consideration.

This search resulted in a list of six studies, summarized in Table 1. For each data set a new curation was made and stored in the InSilico DB to make it trackable. These curations contain the Disease keyword with control and lung cancer as keywords and are used as such through the rest of this paper.

### 2.2. Identification of Differentially Expressed Genes (DEGs)

A very important application of microarray studies is the identification of genes that are consistently and significantly differentially expressed in one group of samples compared to another, according to a target biological variable of interest. These genes are called informative genes, biomarkers, or differentially expressed genes (DEGs). Many methods and approaches to find DEGs exist and here we opted for the R/Bioconductor limma package [18]. Recent and detailed overviews of possible alternative methods can be found in [19, 20].

After applying limma we call every gene significantly differentially expressed if

- it has an adjusted *P* value lower than 0.05;
- it has a log fold change higher than 2.

In order to ensure the robustness of the found DEG lists and to encounter false-positive discoveries we implemented an extra resampling step on top of the limma method. In each iteration, we arbitrarily keep 90% of the samples and apply limma to obtain a DEG list fulfilling the two above-mentioned criteria. After *n* resampling iterations we obtained *n* different DEG lists and our final DEG list will be the intersection of those lists. Taking the intersection might seem rather strict but we empirically confirmed a convergence of the number of DEGs after around 50 resampling iterations, depending on the quality of the study. A resampling iteration size *n* of 100 was used for all experiments.

### 2.3. Experimental Setting

The general workflow for both meta-analysis and merging approaches was already visualized in Figure 1.

For the meta-analysis approach (see Figure 1(a)), we first obtain a robust DEG list—as described above—for each of the six studies individually and then combine the results by taking the intersection of those DEG lists. This final list of DEGs will contain all genes that were found to be informative across all studies consistently.

For the merging approach (see Figure 1(b)), we first merge all six studies into one global data set using the following batch effect removal methods: NONE (no batch effect removal), BMC (batch-mean centering, [21]), COMBAT (empirical bayes, [22]), DWD (distance-weighted discrimination, [23]), and XPN (cross-platform normalization, [24]). Then we applied to each merged data set the same procedure to find robust DEGs. All methods are implemented and documented in the inSilicoMerging R/Bioconductor package [25]. More information on merging through the removal of batch effects can be found in [26].

## 3. Results and Discussion

### 3.1. Meta-Analysis Approach

We first look at the results of the meta-analysis approach by looking at the number of DEGs obtained from the individual data sets as listed in Table 2. In the second column the number of DEGs without using resampling is shown, followed by the number of DEGs after applying resampling as explained in the previous section. We notice that using this resampling strategy leads to a decrease in the number of DEGs for all data sets (ratios between 60 and 80% depending on the specific data set). The rationale behind this extra validation step for the biomarkers discovery is the fact that a stable biomarker should be identified even by making small perturbations in the data set, caused by removing systematically random samples.

Another observation that can be made is the higher number of DEGs for the last three data sets. This difference is probably due to the difference in platform: GPL96 for the first three studies and GPL570 for the last three studies; see Table 1. Since the latter platform has more than 7000 genes more than the former platform, a higher chance of finding DEGs is obvious. Also note from Table 1 that the average sample size for platform GPL96 is around 74, while for platform GPL570 it is around 122; this also can have a minor effect on the robustness of DEGs.

The final list of DEGs in the meta-analysis approach can be obtained by taking the intersection of all single-study DEG lists. This list of 25 genes consists of genes that are consistently differentially expressed in all six studies and can be considered as the most promising list of biomarkers for lung cancer, based on our input data. This list can be found in Supplementary Information (S1) available online at http://dx.doi.org/10.1155/2014/345106.

### 3.2. Merging Approach

In the merging approach, all six data sets are first *merged* into one global data set and thus only one DEG list is finally retrieved. Within this study we applied six different batch effect removal methods resulting in six different lists of DEGs. The results are presented in Table 3 by listing the number of DEGs found for every batch effect removal method. We still notice a need for resampling as it clearly helps to remove false positives, although the difference in number of DEGs with and without resampling is less prominent than in the meta-analysis approach (ratios between 85 and 89% for the different batch effect removal methods).

As a first remark from Table 3 we can observe a relatively low impact of using batch effect removal for this particular study. With the exception of the XPN method, the methods of BMC, COMBAT, and DWD are not able to find more DEGs than when no batch effect removal at all is performed. However, the six lists have 102 genes in common, which is quite a significant result. The final list of DEGs in the merging approach is obtained by taking the intersection of all batch effect removal methods. This list of 102 genes can be found in Supplementary Information (S2).

**Figure 2:**
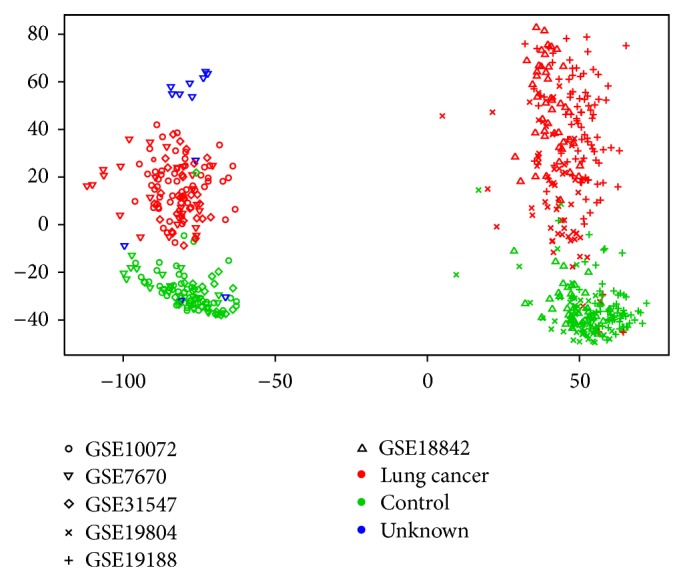
Multidimensional scaling (MDS) plot of the merged data set with no batch effect removal. Samples are colored based on the target biological variable of interest and the different symbols correspond to the individual studies. The figure is generated using the plotMDS function from the inSilicoMerging R/Bioconductor package [25].

Similar results of NONE and the other batch effect removal methods represent a surprising result that we investigated more in detail since there are clearly batch effects present, as is demonstrated in a multidimensional scaling plot of the merged data set with no batch effect removal (NONE) in Figure 2. From this MDS plot we can see that the biggest source of variation is rather technical than biological since all samples are clustered in two groups corresponding to the two different platforms they were assayed on. It could be expected that this undesirable effect would influence the discovery of DEGs. Based on our results this is however not the case and the explanation lies in the fact that MDS plots provide a *global* view on the data, while the identification of DEGs is more based on local effects, that is, the specific expression of one gene in certain conditions. The explanation for this apparently paradoxical result is in the fact that not all genes are affected by batch effect removal in the same way. Genes which are differentially expressed in individual studies could still remain differentially expressed after data merging with no batch effect correction, if their expression is not much affected by batch effects. Moreover, even if a gene is affected by batch effects we observed that the *difference* between the two modes or conditions of the gene (in our case control versus lung cancer) is almost always preserved over all samples of the merged data set.

**Figure 3:**
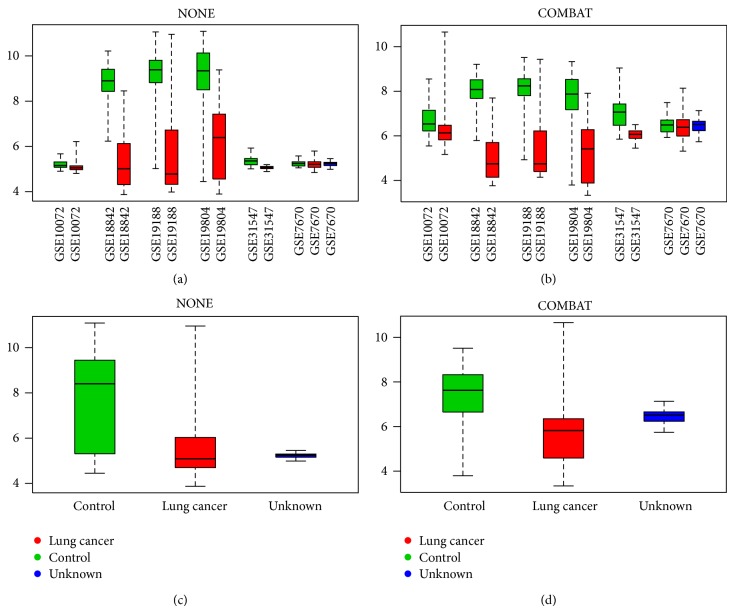
Different boxplots for ADRB1 gene. On the left we have two boxplots for the merged data set without batch effect removal (NONE) and on the right for the merged data set with batch effect removal (COMBAT). All boxplots are grouped and colored based on the target biological variable of interest; the boxplots on top are further grouped per original data set. The figure is generated using the plotGeneWiseBoxPlot function from the inSilicoMerging R/Bioconductor package [25].

To illustrate the local effect of batch effect removal methods we will inspect two genes in detail. First, gene ADRB1 is one of the genes that was only identified as a DEG if no batch effect removal was applied, raising the question if batch effect removal method is distorting the biological signal of this gene. We can look at the boxplots of this specific gene in Figure 3. On the top left plot we can notice that this gene is only differentially expressed in three studies and those three studies are from the same GPL570 platform. For the other studies from the GPL96 platform, the situation is completely different with an almost stable expression of the ADRB1 gene. The difference in expression in the three studies is however big enough to bias the global expression as being differentially expressed (FC > 2), as can be seen in the bottom left plot. If we apply batch effect removal, all samples from both platforms are brought closer together, thereby decreasing the influence of the differential expression of platform GPL570. This results in a global expression that is not differentially expressed anymore (FC < 2) see bottom right plot. From one point of view COMBAT (arbitrarily chosen, other batch removal methods are similar) indeed removes a biological relevant signal that is present in the data, or at least part of the data, but one can argue that this signal is not consistent across all individual studies and can be due to a technical, platform-dependent artifact.

**Figure 4:**
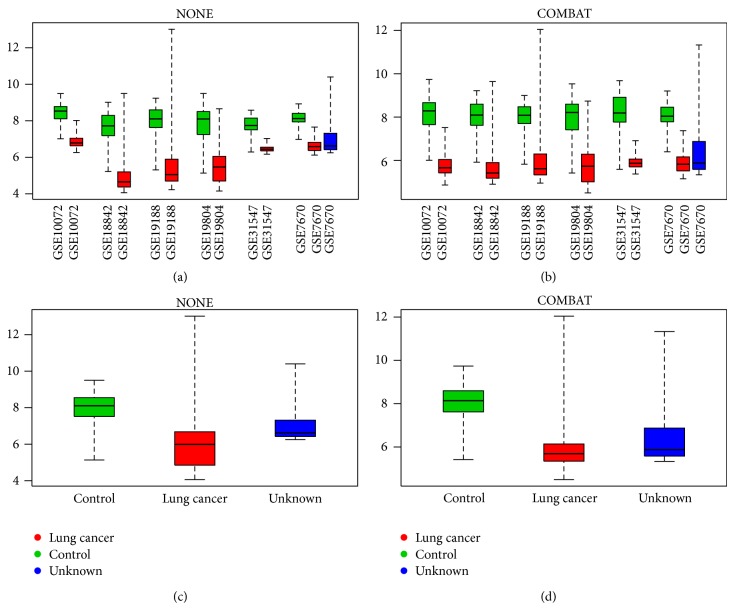
Different boxplots for LRRN3 gene. On the left we have two boxplots for the merged data set without batch effect removal (NONE) and on the right for the merged data set with batch effect removal (COMBAT). All boxplots are grouped and colored based on the target biological variable of interest; the boxplots on top are further grouped per original data set. The figure is generated using the plotGeneWiseBoxPlot function from the inSilicoMerging R/Bioconductor package [25].

We also investigate the gene LRRN3 which, in contrast, was only identified as DEG if batch effect removal was applied. If we compare the top left and top right plots from Figure 4 we can see that COMBAT (again arbitrarily chosen, other batch removal methods are similar) nicely removes the batch effect between the different studies for this gene and creates a clear and consistent differential expression profile across all samples. This leads to a situation in which this gene is labeled as differentially expressed by the COMBAT method, but not by the NONE method since it, just slightly, fails in the log fold change requirement. In this case, instead of a technical artifact, it is actually the batch effect that distorts the global expression profile of the LRRN3 gene.

## 4. Conclusion

Both meta-analysis and merging approaches are able to find differentially expressed genes (DEGs) consistently expressed in all individual data sets. In both approaches a resampling or bootstrapping framework is needed to avoid false positives and to ensure robust gene lists.

Although batch effects were clearly present when merging the different data sets, they were not hindering the identification of DEGs. This surprising finding is however not generalizable to the clustering or classification of tasks since it is very depending on the specific application.

If we compare the final DEGs for the meta-analysis approach with the list obtained in the merging approach we can conclude that significantly more DEGs are identified through merging. Moreover, all 25 identified DEGs through meta-analysis are also identified in the merging approach. Most genes in both lists were scanned in the literature and showed to play a role or at least be involved in the development of lung cancer and can be further validated and used in clinical applications.

## Supplementary Material

In the Supplementary Information two lists of genes that are differentially expressed can be found. The first list (S1) contains 25 genes and is the result of our meta—analysis approach, i.e. the genes are consistently differentially expressed in all six studies. The second list (S2) contains all the 102 genes that are differentially expressed after merging and batch effect removal. All genes in both lists can be considered as promising biomarkers for lung cancer.

## Figures and Tables

**Table 1:** List of six publicly available lung cancer microarray data sets used in this application.

Data set	Platform	No. of genes	No. of samples (control/cancer)	Reference
GSE10072	GPL96	12718	**107** (49/58)	Landi et al. [13]
GSE7670	GPL96	12718	**66** (27/27)	Su et al. [14]
GSE31547	GPL96	12718	**50** (20/30)	—
GSE19804	GPL570	19798	**120** (60/60)	Lu et al. [15]
GSE19188	GPL570	19798	**156** (65/91)	Hou et al. [16]
GSE18842	GPL570	19798	**91** (45/46)	Sanchez-Palencia et al. [17]

Total			**590** (312/266)	

**Table 2:** Number of differentially expressed genes (DEGs) for all individual data sets. The final result of this meta-analysis case is the intersection of the different lists in the last column.

Data set	No. of DEGs^(i)^	No. of DEGs	No. of DEGs^(ii)^
		(resamp.)	(intersection)
GSE10072	90	74	25
GSE7670	79	52	
GSE31547	67	43	
GSE19804	158	109	
GSE19188	351	284	
GSE18842	499	398	

^(i)^Number of DEGs found on the complete data set without resampling. ^(ii)^Number of DEGs in the intersection of the DEG lists of all single data sets after using resampling.

**Table 3:** Number of differentially expressed genes (DEGs) for all merged data sets.

BERM^(i)^	No. of DEGs^(ii)^	No. of DEGs	No. of DEGs^(iii)^
		(resamp.)	(intersection)
NONE	131	112	102
BMC	124	109	
COMBAT	125	110	
DWD	125	111	
XPN	143	123	

^(i)^BERM: batch effect removal method. ^(ii)^Number of DEGs found on the complete data set without resampling. ^(iii)^Number of DEGs in the intersection of the DEG lists for all batch effect removal methods after using resampling.
